# Colchicine-Induced Acute Myopathy: Case Study From Saudi Arabia

**DOI:** 10.7759/cureus.20290

**Published:** 2021-12-09

**Authors:** Moussa Al Megalli, Shahid Bashir, Hanaa Qadah, Omar Ameen, Talal M. Al-Harbi

**Affiliations:** 1 Neurology, Heraa General Hospital, Makkah, SAU; 2 Neuroscience Center, King Fahad Specialist Hospital, Dammam, SAU; 3 Neurology, King Fahad Specialist Hospital, Dammam, SAU

**Keywords:** neuroinflammation, colchicine associated myopathy in saudi arabia, myopathy, drug-induced myopathy, colchicine induced myopathy

## Abstract

Colchicine-induced myopathy has been described in patients with chronic renal failure and patients who are using a concomitant drug like a statin. However, pure myopathy caused by colchicine has never been reported in Saudi Arabia. A 64-year-old patient received colchicine for his gout arthritis disease and developed upper and lower limb weakness. He had a proximal weakness, and his muscle enzymes were very high. Furthermore, the needle electromyography (EMG) examination showed abundant fibrillations, myotonic discharges, and myopathic motor units. Two weeks after colchicine cessation, his weakness improved dramatically with normalization of creatine kinase (CK) and disappearance of myotonic discharges in the repeated EMG. This is the first case in Saudi Arabia that showed colchicine-induced myositis. The local clinicians' community needs to be aware of this rare side effect, as clinical suspicion is the most important diagnostic clue and the only effective treatment is the termination of colchicine.

## Introduction

Colchicine is derived from the corms of Colchicum autumnale and used therapeutically for the treatment of various diseases such as gout, familial Mediterranean fever (FMF), and Behcet's disease for a long time [1-3]. In 1996, the British National Formulary reported a myotoxicity adverse reaction [2]. Although its clinical benefits are well-recognized, colchicine has been associated with gastrointestinal upset, bone marrow suppression, and liver and kidney impairment, particularly during toxic dosages. Colchicine-induced myopathy is an autophagic, vacuolar myopathy that occurs as a rare complication of treatment with colchicine [1]. The onset of colchicine-induced myopathy is commonly related to chronic renal failure or associated with the use of high doses and other myotoxic medications [4]. However, some cases develop colchicine myopathy without statin or renal insufficiency, which raises the possibility of genetic predisposition [5].

In this case, acute myopathy was induced by the administration of colchicine therapy alone over a short period. The resolution of our patient's symptoms and normalization of his creatine kinase (CK) ensured after cessation of colchicine confirmed the diagnosis and led to our patient's recovery.

## Case presentation

A 64-year-old Saudi gentleman presented to the emergency department with acute and progressive upper and lower limb weakness suffering from two weeks. His actual problem started four weeks earlier when he developed severe joint pain and swelling in his right big toe. It was thought to be acute gout arthritis, and he had been given 0.6 mg colchicine three times a day. Subsequently, a remarkable improvement in his joint pain and mobility was observed. Two weeks later, while taking colchicine, he started to have a painless proximal weakness, primarily affecting his legs, causing difficulty stabilizing his gait and rising from a chair and climbing stairs. His past history was unremarkable for any chronic illness. He does not smoke or consume alcohol.

On physical examination, we found him with a high blood pressure of 173/90 with no fever, a body temperature of 36.9°C, and an average pulse rate. The neurology examination was remarkable for symmetrical, more proximal than distal, muscle weakness in the upper and lower limb, hip flexion 2/5, knee extension 3/5, foot dorsiflexion 4/5, shoulder abduction 2/5, elbow flexion and extension 3/5, and wrist flexion and extension 4/5 on the Medical Research Council's Scale (MRC). Otherwise, his cranial nerves, deep tendon reflexes, sensory and coordination examination were normal. He had no skin rash or lymphadenopathy.

Initial laboratory investigations revealed CBC with hypochromic microcytic anemia Hb of 10.1 (13-17 gm/dL), low Na 127 (135-145 mmol/L), K 4.57 (3.5-5) mmol/L, creatinine 0.96 (0.8-1.3) mg/dL, urea 2 (1.2-3) mmol/L, corrected immediately with hydration. Creatinine phosphokinase (CPK) was high (2974 (25-200) U/L), normal glycated hemoglobin (HbA1C) 5.4%, thyroid stimulating hormone (TSH) 3.12 (0.5-5) mIU/L, and T4 17.54 (4.9-11.7) mg/dL. Cerebrospinal fluid (CSF) analysis revealed normal cell count with normal CSF protein and sugar, and serum tumor markers were negative.

**Figure 1:**
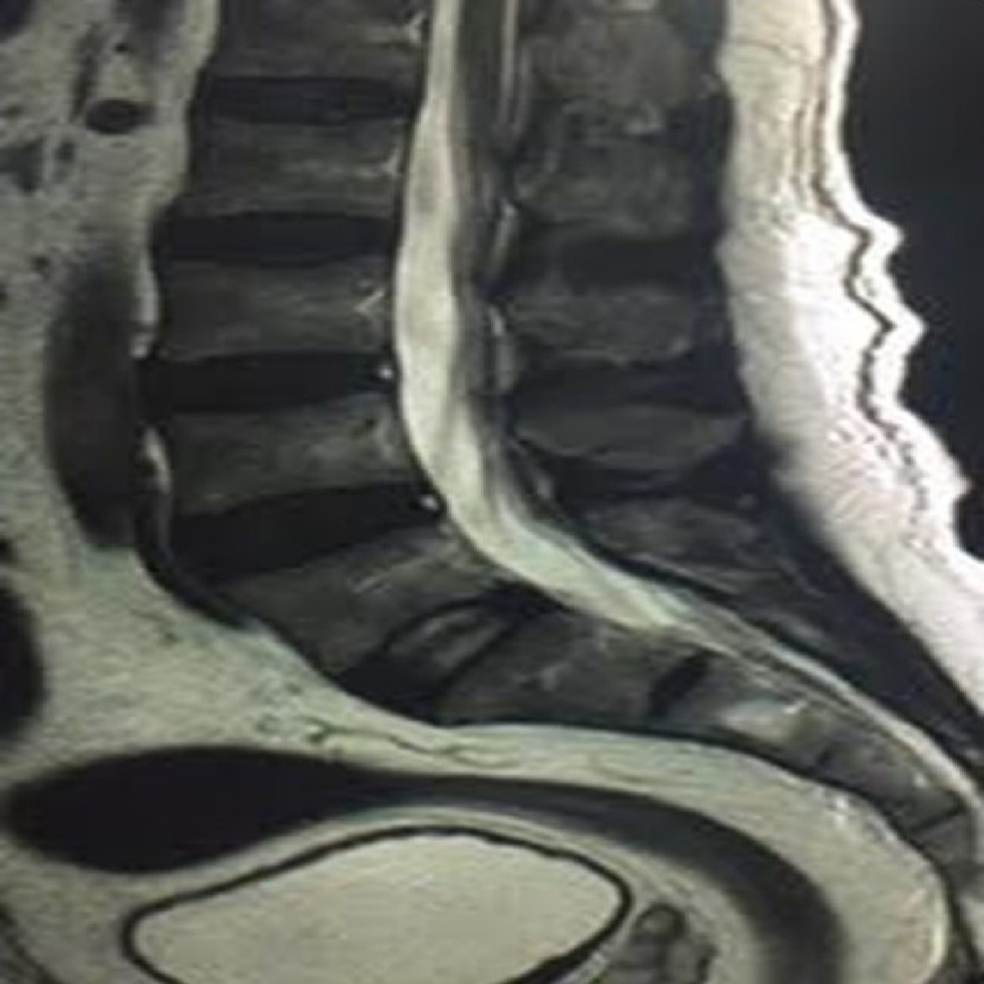
T2 weighted image for the lumbosacral spine showing multiple degenerative changes with no radiculopathy

MRI of the lumbosacral spine was unremarkable (Figure 1).

The nerve conduction study (NCS) showed a mild drop in compound action muscle potentials (CMAPs) of the tibial nerve from the abductor hallucis and the common peroneal nerve from the extensor digitorum brevis with normal distal motor latency and conduction velocity (Table 1).

**Table 1:** Sensory nerve conduction study showing normal distal latency, amplitude, and conduction velocity in the upper and lower limbs Lat: Latency. Amp: Amplitude. CV: Conduction Velocity. ms: Millisecond. (m/s): Meter/ second. uV: Microvolt. Dig: Digit.

Nerve	Peak Lat	Amp	CV
	ms	uV	m/s
Median Sensory Right			
Wrist - Dig II	3.50	81.6	50.2
Ulnar Sensory Right			
Wrist - Dig V	2.17	64.9	61.7
Sural Sensory Left			
Mid. Lower Leg - Ext Saph	3.79	21.1	46.5
Sural Sensory Right			
Mid. Lower Leg - Ext Saph	4.00	27.8	45.0
Superficial Peroneal Sensory Left			
Lateral Malleolus - Ankle	2.81	18.6	47.4
Superficial Peroneal Sensory Right			
Lateral Malleolus - Ankle	2.52	20.6	50.0

On the other hand, sampling different proximal muscles with needle examination demonstrated extensive fibrillations, positive sharp waves, abundant myotonic discharges with myopathic pattern motor units, and increased recruitment patterns (Table 2).

**Table 2:** Electromyography findings Fib: Fibrillation Potentials. PSW: Positive Sharp Waves. Myot: Myotonia. Amp: Amplitude. Dur: Duration. Poly: Polyphasic. +: 1+. ++: 2+. +++: 3+. Med: Medial Head. MUAP: Motor Unit Action Potentials.

	Insertion							MUAP
Muscle	Insertion Activity	Fib	PSW	Myot	Amp	Dur	Polyphasics	Recruit
Right Deltoid medial	Increased	++	+	+++	Low	Short	+++	Increased
Right Biceps	Increased	++	++	+++	Low	Short	+++	Increased
Left Biceps	Increased	++	++	+++	Low	Short	+++	Increased
Left Triceps	Increased	++	++	+++	Low	Short	+++	Increased
Right Triceps	Increased	++	++	+++	Low	Short	+++	Increased
Right Vastus Medialis	Increased	++	++	+++	Low	Short	+++	Increased
Left Vastus Lateralis	Increased	++	++	+++	Low	Short	+++	Increased
Left Gastroc Med	Increased	++	++	++	Low	Short	+++	Increased
Right Gastroc Med	Increased	+++	+++	++	Low	Short	+++	Increased
Left Tibialis Anterior	Increased	+++	+++	+	Low	Short	+++	Increased
Right Tibialis Anterior	Increased	+++	+++	+	Low	Short	+++	Increased

Following discontinuation of colchicine by two weeks, the patient started to improve clinically, and his creatine phosphokinase (CPK) declined to an average level (200) U/L. Furthermore, repeated needle examination demonstrated complete resolution of the fibrillation potential and myotonia with the recovery of the motor units. At the end of the second week, his proximal muscles became 4+/5, and the patient was discharged home, walking independently.

## Discussion

We believed that colchicine therapy was the cause of acute myopathy in our patient based on the following facts: the patient's weakness started within weeks of beginning colchicine therapy. At the time of his presentation, he had strong evidence of active myopathy based on symptoms and supported by the elevated muscle enzymes and myopathic EMG picture of the proximal muscles. Rather than restoring his muscle strength, there was normalization of muscle enzymes and electrophysiological recovery in the needle examination following the discontinuation of colchicine. Another diagnostic possibility was considered, like polymyositis. However, in that case, he would not have recovered without using steroids or other immunosuppressive medications. With this subacute generalized weakness, the diagnosis of acute inflammatory demyelinating polyneuropathy (AIDP) was also entertained. Nevertheless, it was ruled out by finding a normal CSF analysis and the lack of demyelinating features in the NCS like prolonged F wave and neurogenic motor units in the needle examination, which did not exist. Negative tumor markers also excluded paraneoplastic neuropathy.

We searched the PubMed electronic database for literature, using the keywords "colchicine-induced myopathy"; and "neuromyopathy in Saudi Arabia." We identified 114 articles in which colchicine-induced neuromyopathy was reported worldwide [6]; none were from Saudi Arabia. Myotoxicity is usually more severe and more common than neurotoxicity.

Colchicine-associated myopathy risk increased with the presence of other co-morbid conditions like chronic renal failure, hepatic failure, coadministration of drugs like simvastatin, tacrolimus, cyclosporine, erythromycin, and antifungal medications [4,6-8]. Colchicine is a known tubulo-toxin because it inhibits microtubule polymerization by binding to the α and β monomers of tubulin. The mechanism by which colchicine causes myopathy is largely unknown. However, it is known for causing painless vacuole myopathy, affecting the microtubular network, and transport causes vacuolation [9-10]. Myopathy is more common and profound than neuropathy. Moreover, acute rhabdomyolysis has been reported in association with colchicine therapy [11].

One of the important factors in colchicine's metabolism is ABCB1 (also known as multidrug resistance protein 1 or p [1] glycoprotein, encoded by ABCB1) [12]. There is some indication that genetic variation in ABCB1 may contribute to the variability in colchicine's effectiveness for familial Mediterranean fever [13]. Gupta et al. reported two cases with colchicine-induced myopathy muscle pathology, sequencing of genetic polymorphisms previously associated with altered colchicine metabolism, suggesting genetic variants polymorphism of ABCB1, particularly the homozygous c.3435T allele, may have a contributing factor in developing myopathy associated with colchicine [5]. Future study of the ABCB1 polymorphism or other genetic factors in a larger series of colchicine myopathy patients was suggested by the authors [5].

## Conclusions

We present the first reported case in Saudi Arabia that demonstrates colchicine-induced myopathy, which is considered an infrequent adverse event of this critical drug. However, clinicians should be aware of this rare side effect, as clinical suspicion is the most important diagnostic clue, and the only effective treatment is the termination of colchicine. Moreover, the genetic susceptibility to colchicine-induced myopathy is a potential for future study to identify people who are more prone to develop this rare side effect.
